# Wishes and Needs of Nursing Home Residents: A Scoping Review

**DOI:** 10.3390/healthcare10050854

**Published:** 2022-05-06

**Authors:** Roxana Schweighart, Julie Lorraine O’Sullivan, Malte Klemmt, Andrea Teti, Silke Neuderth

**Affiliations:** 1 Institute of Gerontology, University of Vechta, 49377 Vechta, Germany; andrea.teti@uni-vechta.de; 2 Charité—Universitätsmedizin Berlin, Corporate Member of Freie Universität Berlin, Humboldt-Universität zu Berlin, and Berlin Institute of Health, 10117 Berlin, Germany; julie.osullivan@charite.de; 3 Faculty of Applied Social Sciences, University of Applied Sciences Würzburg-Schweinfurt, 97070 Würzburg, Germany; malte.klemmt@fhws.de (M.K.); silke.neuderth@fhws.de (S.N.)

**Keywords:** quality of life, well-being, long-term care, person-centered care, older adults, needs assessment, needs fulfillment, healthy aging

## Abstract

Falling birth rates and rising life expectancy are leading to global aging. The proportional increase in older people can be observed in almost all countries and regions worldwide. As a result, more people spend their later years in nursing homes. In homes where person-centered care is implemented, residents report greater satisfaction and quality of life. This approach is based on the wishes and needs of the residents. Therefore, the purpose of this scoping review is to explore the wishes and needs of nursing home residents. A scoping review of the literature was conducted in which 12 databases were systematically searched for relevant articles according to PRISMA-ScR guidelines. Both quantitative and qualitative study designs were considered. A total of 51 articles met the inclusion criteria. Included articles were subjected to thematic analysis and synthesis to categorize findings into themes. The analysis identified 12 themes to which the wishes and needs were assigned: (1) Activities, leisure, and daily routine; (2) Autonomy, independence, choice, and control; (3) Death, dying, and end-of-life; (4) Economics; (5) Environment, structural conditions, meals, and food; (6) Health condition; (7) Medication, care, treatment, and hygiene; (8) Peer relationship, company, and social contact; (9) Privacy; (10) Psychological and emotional aspects, security, and safety; (11) Religion, spirituality; and (12) Sexuality. Nursing home residents are not a homogeneous group. Accordingly, a wide range of needs and wishes are reported in the literature, assigned to various topics. This underscores the need for tailored and person-centered approaches to ensure long-term well-being and quality of life in the nursing home care setting.

## 1. Introduction

Declines in fertility rates and increases in life expectancy are leading to global population aging. The proportional growth of older people in almost all countries and regions worldwide supports this premise [1]. This demographic change is considered one of the most significant social transformations of the 21st century by the United Nations [1].

In parallel, the number of older people in need of care is also increasing. In Germany, for instance, more than 800,000 people were receiving full inpatient care in a nursing home (NH) at the end of 2019 [2]. These trends bring forth the projection that by 2030 there will be a demand for 1.3 million NH places [3].

Quality of care and residents’ quality of life is still suboptimal in some NHs. Efforts are being made to implement a culture change to improve the quality of the homes [4]. This change is intended to shift away from a focus on physical care and a standardized approach to person-centered and individualized care. The person-centered approach is holistic and views residents as individuals. Respectful discourse with the resident is fundamental to promote care oriented to resident needs and values [4,5].

In NHs where person-centered care is implemented, residents are more satisfied with the quality of care and service. Life satisfaction, overall satisfaction, and quality of life are also higher among residents of homes with person-centered care [6,7]. Implementation of person-centered care for NH residents requires a foundation built on the recognition of their wishes and needs. Thus, fulfilled wishes and needs lead to greater life satisfaction [8]. Consequently, identifying and addressing these can improve quality of life and care for NH residents. Therefore, the purpose of this scoping review is to provide an empirical overview of the range of wishes and needs of NH residents. Previous reviews have already assessed the wishes and needs of older people. However, these have focused either on people receiving home care [9] or exclusively on NH residents with dementia [10,11].

## 2. Methods

We conducted the present review in accordance with the framework proposed by Peters et al. from the Joanna Briggs Institute (JBI) [12]. The framework includes the following nine steps:

(1) Defining and aligning the objective and question; (2) Developing and aligning the inclusion and exclusion criteria with the objective and question; (3) Describing the planned approach to evidence searching, selection, data extraction, and presentation of the evidence; (4) Searching for the evidence; (5) Selecting the evidence; (6) Extracting the evidence; (7) Analyzing of the evidence; (8) Presenting the results; and (9) Summarizing the evidence in relation to the purpose of the review, making conclusions, and noting any implications of the findings.

We registered the protocol for the study in advance on the Center for Open Science (OSF) in October 2020 [13]. The review process was conducted and the findings were documented and reported according to the Preferred Reporting Items for Systematic reviews and Meta-Analyses extension for Scoping Reviews (PRISMA-ScR) checklist [14].

### 2.1. Inclusion and Exclusion Criteria 

Beforehand, the authors defined the inclusion and exclusion criteria jointly and recurrently reviewed these during the process. Empirical qualitative and quantitative articles that included wishes and needs of people living permanently in a NH were included in the review. Both articles with self-reported and proxy-reported wishes and needs were considered. On average, residents were required to be 60 years of age or older. In addition, we considered only articles published between the years 1990 and 2020 written in English or German. Of interest were studies that included the constructs “wishes” and/or “needs”. To assure this, a definition was established in advance. Generally speaking, defining wishes and needs includes the initiation of a desire, fulfillment, and a positive resulting effect. In detail, wishes and needs are:

Any desire or craving that the person subjectively feels within him- or herself, whether this is material or immaterial, for change or preservation, already fulfilled or still unfulfilled, realistic or unrealistic, current or future, more or less urgent. The fulfillment of this desire causes a positive effect within the person. This positive effect can be related to the quality of life, satisfaction, self-image, autonomy, and any other aspect of the person’s life.

We excluded articles addressing people who were, on average, under 60 years old or people who did not live in a NH. We only included original empirical studies that had already been published in a journal in order to ensure, as far as possible, that only relevant and high-quality studies were considered. Accordingly, gray literature, conference proceedings, books, book chapters, reviews, and dissertations were rejected.

### 2.2. Searching for the Evidence

We explored relevant journal articles in 12 databases (PubMed, PsycINFO, CINAHL, LIVIVO, Embase, Cochrane Library, GeroLit, SCOPUS, AgeLine, SowiPort, WiSo, and Psyndex) during August and September 2020. A search strategy was developed for each database. Table 1. Search Terms contains the specific search terms in English and German.

### 2.3. Selecting the Evidence 

**Figure 1:**
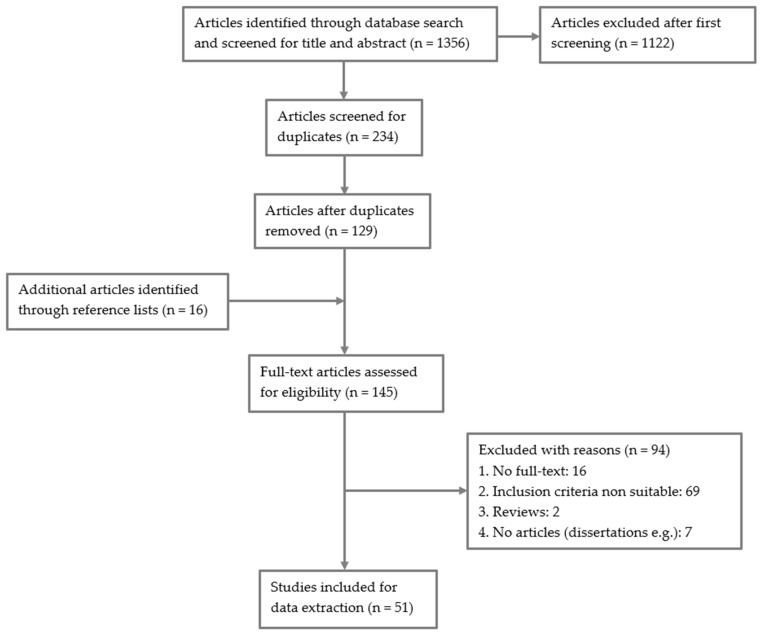
Search flowchart following PRISMA guidelines.

The article selection went through several phases. Three authors completed the database search separately, so each searched four databases. The studies identified by the database search were first screened by title and abstract by one author regarding relevance and fulfillment of the inclusion criteria. The remaining articles were sequentially screened for duplications and removed if necessary. In a final step, each article was screened for relevance and compliance with the inclusion criteria independently by two authors based on the full text. In case of discrepancies between the reviewers, the third author was consulted. Figure 1. Search flowchart following PRISMA guidelines illustrates the details of the article search.

Of the 1356 articles initially discovered through the database search, 51 articles met the inclusion criteria. 

### 2.4. Extracting the Evidence

The included articles underwent a structured data extraction by three authors capturing essential study information. These include author(s), year, title, journal (number and page), country, sampling strategy, sample characteristics, design and method, data analysis, and relevant results. The reviewer’s name, who performed the extraction, and the data extraction date were also recorded. For quantitative studies, the five most frequently identified wishes and needs were extracted in each case to ensure that the most relevant outcomes were included. 

### 2.5. Analysis of the Evidence 

After extraction, the relevant results (which relate to the research question and thus to the wishes and needs of the NH residents) were analyzed using thematic analysis following Braun and Clarke [15]. We developed a category system inductively on the 51 included studies following this approach. Three authors performed the thematic analysis and synthesis. Agreement between the authors led to final system revisions, checks for consistency, and the decision to group individual needs in their context and assignment to the top themes.

## 3. Results

### 3.1. Characteristics of the Included Articles

The final analysis includes 51 articles consisting of 28 studies with a quantitative study design, 20 with a qualitative design, and three mixed-methods studies. Of the 28 quantitative studies, 26 were cross-sectional surveys and two were longitudinal. Questionnaires were used to collect data on 26 studies, one of which was a cluster randomized controlled trial (cRCT). The Camberwell Assessment of Need for the Elderly questionnaire (CANE) [16] was used for 10 studies, while four studies used the Preferences for Everyday Living Inventory-Nursing Home questionnaire (PELI-NH) [17]. Of the 20 studies with qualitative designs, 19 were cross-sectional surveys. Sixteen studies used interviews to generate data, one study used the focus group method, and three collected data in interviews and focus groups. All three mixed-methods studies had a cross-sectional design and used both questionnaires and open-ended questions. Thirty-five of the 51 studies assessed self-reported wishes and needs, while 12 interviewed both residents and others, including relatives and family members, caregivers, and nursing assistants. In contrast, four studies surveyed only proxies. Caregivers, volunteers, public guardians, relatives, family members, and non-nursing staff were interviewed. Table 2. Study Summary illustrates the summary of each study.

### 3.2. Wishes and Needs 

As a first step, we present the results of 41 studies on wishes and needs of NH residents, excluding those that used the CANE questionnaire. Subsequently, we present the results of the remaining ten studies that collected data on wishes and needs with the CANE instrument. This separation seemed reasonable, as the CANE questionnaire is the only instrument that explicitly distinguishes between met and unmet needs. Therefore, the separate presentation and summary of the CANE studies provide a comprehensive overview of the results collected with this questionnaire. The wishes and needs found in the 41 studies presented first could be mapped to 12 themes. These are shown in detail in Table 3.

#### 3.2.1. Activities, Leisure, and Daily Routine

The need to make the day active and momentous has been addressed in several studies [27,28,38,46,49,58,60]. Accordingly, wishes for meaningful, person-specific, enjoyable, social, and recreational activities were mentioned [27,28,38,46,60]. Residents like to practice their hobbies and consider activities on special occasions and events as important [27,38]. Various pursuits and leisure activities that residents like to do could be classified under this theme: Reading, listening to music, having contact with animals, keeping up with the news, spending time outside, doing activities outside the NH, playing games, partying, tea-time, gardening, helping others, doing crafts, and spending time with others [27,38,46,49,58]. In addition to the need for specific activities, a general wish for a varied life with diverse offerings and activities was also mentioned [60,62], in which residents can experience self-sufficiency [49].

#### 3.2.2. Autonomy, Independence, Choice, and Control

Moving into an NH can result in a loss of autonomy and independence. Over half of the 41 studies [20,22,25,26,28,30,31,32,33,35,36,38,39,49,50,52,57,58,60,62,63] demonstrate that it is essential for residents to do things for themselves, to have a say in decisions, and to maintain their autonomy to the greatest extent possible. In various studies, NH residents described an experienced dependence and a wish to gain more autonomy and independence: “The stroke nurse who was to do the swallowing test never came. She was to sign me off for swallowing so that I could eat bread… You see I am very determined to be as independent as I can be? I would love to be able to walk to the toilet on my own” [52]. Residents reported a wish to make decisions for themselves or to be involved in the decision-making process and that this is central to their well-being and quality of life [60,62]. The need to have a say relates to both day-to-day issues and far-reaching decisions. For example, residents wish to have control over daily concerns such as deciding when to get up and go to bed [28,38,39], what clothes to wear [38,58], what and when they eat [28,39,49,63], how they spend their day [49], who they share a room with [39], and whether they participate in social activities [49]. Residents also want to make their own decisions on issues related to hygiene and care routines, including bathing and showering type, how often to bathe or shower, and oral hygiene [35,36,38,39,63]. Control over medical matters is highly important to many residents. For instance, residents would like to have a choice regarding how often and which physician they consult [35,39]. Residents are concerned about their future and would like to make advance directives and living wills. According to one study [67], over one-third of residents have a written advance directive, i.e., either an advance directive, or a living will, or a combination of different documents. Residents who already have an advance directive most often want their son or daughter, or a close relative, to act as surrogate decision makers should their own decision-making capacity cease [26,50]. In decisions concerning care, residents wish to determine who has a say for themselves. Some residents wish to make all decisions on their own, but many would also like family members and relatives to have a say, while still others would like staff or the attending physician to make final decisions and hand over responsibility to them [22,25,31,32,35,62].

To maintain a sense of freedom and independence, residents feel the need to regularly leave the NH on their own and independently [39,57,63]: “I tell a member of staff when I leave the NH. This is not a problem. Sometimes I am not back before midnight. I have a key. So, I can come and go whenever I want. That’s great. Because the staff do not have to give a key to the residents” [57]. Some residents want to move out of the NH or want to have control over their own discharge. This is partly based on the need to live in familiar surroundings again, but also on the wish for more self-determination and freedom [33,39,62].

#### 3.2.3. Death, Dying, and End-of-Life

People often move into a NH at a late stage in life, when the issues of dying and death become increasingly important. Residents have different ideas about the end of their lives and dying in the home. NH residents wish not to become bedridden and in need of care in the last phase of life. Furthermore, they wish that their health condition does not deteriorate further allowing for a degree of mobility and activity. Despite impending death, residents want to continue to make plans and be content [40,41]. Contact with family members, friends, relatives, and other confidants, such as nursing staff, or the attending physician, plays an essential role in this phase of life [40,66]. The results show that residents are concerned about discussing the topics of dying and death with familiar people. Residents want to prepare for death and plan for the process of dying and the time after [57]. In addition to a general need to talk about the approaching death, residents are particularly concerned about symptom management, emotional, psychological, and spiritual support, possible counseling services, and funeral issues [27]. One study [32] found that there is often a lack of opportunities to discuss one’s values and needs regarding end-of-life treatment and care with the nursing staff. Resident reactions to such staff discussions vary greatly from unnecessary to a very strong need. Wishes for pain management and more personal and time-intensive care include maintaining personal hygiene and the requirement of additional medical care in the last phase of life [40,56]. There are also clear wishes and needs on the part of NH residents regarding the dying process. In this context, several studies shed light on the context in which people want to die, such as the place of dying, the condition in which they want to die, and the people they would like to have by their side when dying [26,41,50,66,67]. In most cases, residents would like to die in the NH and not be transferred to another facility, such as a hospital. [26,41,50,66,67]. However, needs for passing away at home, in hospice, or in a hospital are also cited [66,67]. Most residents in one study [66] reported wanting to pass away in their sleep (31%). Fewer residents would like to be unconscious or comatose during dying (7%) and a small percentage would like to experience the dying process while conscious (3%). The other residents were not clear at the time of the survey about the condition in which they would like to die or did not make any statement for other reasons. 

The question of end-of-life care also seems to be essential for residents. For example, most residents wish to die in the presence of familiar people, such as relatives, friends, nursing staff, or hospice companions. “That I can cling somewhere,… to any hands…” [41]. Others would rather be alone when the time comes [22,41]. When dealing with dying people, physical closeness, human warmth, support, and respectful, open, and honest communication are of great importance [41,66]. Medical and nursing factors are also central. Residents do not want to suffer pain and thirst during the dying process and want to be able to breathe comfortably [22,40,41,56,66]. Many residents do not want to receive life-sustaining measures, including artificial nutrition, resuscitation, surgery, heart–lung machine, ventilator, or dialysis, during the dying phase [22,40,41,56,66]. However, others want to receive life-sustaining treatment in the event of a life-threatening condition [50]. Residents consider a natural and quick death, which they see as a release, important [22,49].

Spiritual factors also play an essential role when residents face death in a NH. Residents want to die quietly and peacefully, which means that they do not want to be a burden on anyone and want to die without much fuss. They wish for forgiveness and reconciliation, for their mistakes not to be of great relevance in retrospect, and for their loved ones to think back on them positively after their passing [41]. During the dying process, residents feel the need to maintain their dignity and self-respect and to leave the world laughing [66]. 

The wish to die or to actively end life has also been cited in studies [33,57,66]. Three of 18 residents interviewed in the Goodman et al. study [33] want their life to end. Van der Steen et al. [66] found that residents wish to have ways to end life if they feel it is necessary.

#### 3.2.4. Economics

Four of the 41 studies [24,27,60,62] captured residents’ financial wishes and needs. All four studies found a desire for more money or financial support and financial security. Chuang et al. [27] also found that residents feel a need to be able to pay the monthly NH fee. If this cannot be accomplished, residents would be discharged or transferred to another NH with lower standards, which they try to avoid.

#### 3.2.5. Environment, Structural Conditions, Meals, and Food

Studies reported facility-related needs and needs at the structural level, for example, concerning the room occupied [19,20,36,38,46,47,60,62]. Residents wish for a comfortable bed [19], larger [62] and temperature-controlled rooms [36], and the ability to personally furnish the rooms with their own furniture, objects, photos, a television, and a radio [46,60]. Further, needs were expressed for housing facilities that are designed for the elderly and disabled such as the presence of elevators [62]. Clean housing and sanitary facilities are also important to residents. Regarding these, the wish for improvement was mentioned [20,62]. It is also essential that residents can take care of their own belongings and have a way to lock and store smaller items safely [19,36,38]. Other needs related to facility structure include a wish to separate residents with dementia from those without dementia and a wish for more flexible routines. For example, residents would like more flexibility in the timing of taking pills [62]. Culinary care in the NH also plays an essential role for residents. According to Sonntag et al. [62], residents feel the need for better food that is age-appropriate and not so monotonous. In addition, residents want to decide what food they get, how much of it, and whether they eat according to a recommended diet. Some wish for more traditional food to be offered and to take meals at their leisure, without time stress, at set times of the day, and with patient and respectful assistance if necessary [47]. Housen et al. [38] reported that it is important for residents to have snacks available at their convenience in the NH. 

#### 3.2.6. Health Condition

An inability of older people living alone with deteriorating health and physical condition often requires a transition to NH. Thus, the issue of health is of high importance for these NH residents. Most common among this theme was the need to maintain and improve health or to prevent a decline in health [33,49,57,60,61,62]. In this context, maintaining both cognitive and physical health status is of high importance. The abilities are seen as a prerequisite for enjoying the last years in the NH: “The few years I have left to live, I want to enjoy them. I can still walk, more or less, well around what you can call walking. You don’t need to put me in a chair yet, a wheelchair or one of them frames. Yeah, I do and wash myself and everything” [60]. As the worst imaginable scenario, residents describe their condition as a nursing case: “I have no expectations anymore. The principal thing is not to become a nursing case. I do not want to become an invalid like some of the other residents. I do not want to lose my mind. In this case, I would rather die” [57]. According to Schmidt et al. [61], residents wish to maintain their physical and sensory awareness. 

Additionally, full and honest information about one’s health status is also noted to be essential. While three studies [20,26,60] reported that residents want to be fully informed about health status and, if applicable, fatal diagnoses, Gjerberg et al. [32] found that a small number of residents were unsure whether they might want to receive information of a severe nature or indicated that they did not want to receive information. This is due to fear of harmful consequences, “…that will just leave me thinking. And I would rather not”.

Wishes for mobility or physical activity [57,60,61], for physical comfort [63], and for restful sleep and sleep comfort [58,60,61] were also mentioned under the topic of health condition. 

#### 3.2.7. Medication, Care, Treatment, and Hygiene

Thirteen of the 41 studies [18,19,20,27,30,33,35,37,42,46,49,58,62] addressed needs related to the behaviors or characteristics of nursing staff or care received. For example, residents want to receive care that is good [37,58], humane [62], continuous [37,42], competent, skilled [27,62], affectionate [62], encouraging [42], and professional [19]. According to Bangerter et al. [19], professional care in this regard can be defined as friendly, kind, courteous, emphatic, respectful, and characterized by symmetrical communication. Further, residents want to be perceived as individuals, treated personally and with dignity, and taken seriously [30,37,62]. This includes addressing residents personally by name [19]. They wish staff would reliably take care of them and be concerned about them [18,27,33,35]. Residents feel the need to trust the nursing staff [20] and have a good relationship with them [33]. Sensitivity and motivation on the part of caregivers are necessary to form a trusting relationship possible according to residents [42]. This does not always seem to be guaranteed: “Not too many of them help too much when we’re not well-they don’t have feelings… They are tired-they have to lift me and I’m heavy. If they have a bad day or bad night, they lose the ability to be sensitive to our condition. Sometimes I feel that they take their frustrations out on us. They lack a little sensitivity” [42]. Residents wish they were not treated as if they were a nuisance, a problem case, or a child [46,49]. 

In addition to needs primarily related to nursing staff, residents also reported wishes and needs related to medical care and hygiene. According to different studies [42,58,61] personal hygiene is important to residents. This includes bathing and washing facilities [58], oral hygiene, and regular changing of linens [42]. In one qualitative study with 10 women and 10 men, some women reported a gender-specific need for personal care to be performed by a caregiver who is a woman herself [37]. High-quality medical care includes the use of proper equipment during treatments [20], good skin and wound treatment, expert pain management to prevent discomfort due to physical illness [61], and monitoring for adverse drug reactions [46]. Referring to the study by Michelson et al. [45], residents refuse aggressive medical treatment unless the intervention alleviates pain or results in greater patient comfort or safety. Nakrem et al. [49] and Sonntag et al. [62] found that residents hope to receive more active care in the NH, more therapeutic interventions, more physical therapy, and regular fall prevention by NH staff. To provide more quality of life in the NH, residents wish for more help and support with daily living activities [27,62]. Frustration is reported because this support is not provided by staff without being asked [42]. Residents reported care needs for eating and drinking, excreting, constipation, sleep disturbances, loss of appetite, chronic illnesses (including asthma, arthritis, hypertension), and visual impairment [23,61]: “The constipation has given me piles in that my whole body is affected” [23].

In the study by Levy-Storms et al. [42], excessive cross-boundary support from nursing staff is sometimes reported: “Let me eat (feed myself) with a spoon, like normal people”. This is countered by the reports of residents who experience a lack of individualized and skilled care and attention from NH staff. This is seen as a problem of limited staff capacity, which is why the wish for more staff was mentioned to make the above-mentioned needs and wishes feasible [62]. 

#### 3.2.8. Peer Relationship, Company, and Social Contact

Contact with other people is a central need for many NH residents. While a good and trusting relationship with the nursing staff has already been presented as the basis for humane and personal care, residents name social contacts and friendships as significant for a satisfying life in the NH. Residents described needs for sociability and conversation in their lives [30,62], for human connection [52], for belonging [30], for a good and personal atmosphere in the home [60], for harmony [23], and for meaningful relationships [55]. 

Relationships with other NH residents are highly relevant, as these play a significant role in determining the daily environment. Residents actively choose their contacts in the NH, talking about their experiences in the home, their past lives, and their families. They spend time together and do things together: “I am in touch with Anna. She lives down the corridor. She is lucid, and we can talk. She comes to visit me, and then we talk… and if she gets some sweets, she comes to me [to share] and if I get something she appreciates from my family, then I share it with her” [21]. Residents reported a wish for all residents to live better together [62] and a desire for personal and social relationships with other residents [21,27,28,49,60]. 

In addition to the need for in-home relationships with peer residents, the wish for good relationships with family members, relatives, and friends outside the home was also frequently mentioned. For example, residents would like to maintain family and friendship ties [21,27,28,52,60,63] and spend more time with and are regularly visited by their loved ones [18,20,21,30,35,62]. 

Residents also wish to maintain contact with their former social environment and the community they lived in before moving. Residents do not want to lose connection to their former lives and the world outside the NH [28,49,52,63]: “I like getting out to the town, you know. I just like to see if there is any building going on or what’s happening in the town” [52]. Residents indicate they want to maintain their past relationships and ties because they are identity-building [52]. Ways to maintain a connection to the outside world include: watching television, listening to the radio, reading the newspaper, or sitting at the front door to watch people come and go [63].

#### 3.2.9. Privacy

As important as human contact is, a certain degree of privacy is likewise important. This was shown by seven studies [19,20,27,28,33,38,60]. Residents desire privacy when using the restroom and performing personal hygiene [19,60]. The wish for privacy further includes the need for a private space [60], which residents understand to mean, for example, occupying a single room [28], but also being able to receive visits or make telephone calls in a private setting [38].

Quietness in the NH is also crucial to residents’ privacy. They wish to rest undisturbed [33] and that they are not disturbed by loud noises [60]. 

Residents who inevitably interact with others due to the institutional setting want to spend time alone [60] and consider it important for social and psychological privacy that nursing staff knocks upon entering the room [28]. Cooney et al. [28] found that residents of large facilities particularly complained about a lack of privacy. In some cases, beds are separated only by curtains, which ensures a very low level of quiet and privacy: “You only have a curtain separating you” [28]. 

#### 3.2.10. Psychological and Emotional Aspects, Security, and Safety

Many of the wishes and needs of residents are also in the psychological, emotional, and safety domains. Inner-personal and psycho-emotional needs, for example, were named in the study by O’Neill et al. [52]. Residents wish to have a positive attitude and maintain their own identity, self-efficacy, resilience, and coping strategies. They would like to take each day as it comes and not worry too much about tomorrow. According to Franklin et al. [30] and Schmidt et al. [61], residents want to experience a daily routine, to be able to enjoy the little things in everyday life, and to find a sense of meaning in the NH’s daily routine to experience themselves as part of the environment. It seems essential for residents to have a sense of belonging, to feel understood, and to have a sense of community [60]. Other studies report similar findings [28,61,63]: residents want to be themselves, not lose a sense of self, and be recognized as independent individuals. To ensure this, residents are concerned about their appearance among others. One qualitative study showed that some women want to take care of their appearance. They state that this has a positive effect on their self-expression and self-esteem [28].

Further, having options to do what they want when they are miserable is essential [18,36]. Fundamental to residents is that they feel needed, valued, and welcomed [27]. Schmidt et al. [61] also found that expressing emotions, expressing one’s will, being talked to and touched, as well as touching others are important for residents’ emotional and psychological well-being. NH residents wish for social and emotional support in the home [46] and psychological support for depression, confidence loss, memory loss, anxiety, anger, and irritability [23]. 

A sense of security is also important to residents. They wish to be safe and secure in the NH [49,60,61]. This includes knowing that the home has safety and security measures installed and that residents always have quick access to emergency services [20,49]. Being protected from self-harm and from disturbance by other residents is also part of living safely in an NH [46].

#### 3.2.11. Religion and Spirituality

Religiosity and spirituality play an important role for many residents. For example, they wish to participate in religious ceremonies [27,38,43,58,61]. They want to express themselves religiously in their lives, follow cultural customs, and feel spiritually connected to others [27,38,61,63]: “I can’t go to the Sunday ceremony, but I read the Bible by myself… You will feel consoled after you read it” [27]. Specific activities that residents undertake to meet their religious and spiritual needs are cited by Man-Ging et al. [43]: praying for themselves, reflecting on past lives, turning to a higher presence, and plunging into the beauty of nature. 

#### 3.2.12. Sexuality

One study [48] addressed the sexual needs of NH residents. More than half (51%) of the residents surveyed reported a sexual tension, including more men (65%) than women (41%). In addition, residents reported the following as their most important sexual needs: need for conversation, need for respect, need for tenderness, need for support in any situation, and need for giving and receiving emotional support, by which residents primarily mean empathy and understanding.

#### 3.2.13. CANE Studies

The ten studies that used the CANE questionnaire for data collection are presented separately. The CANE questionnaire covers 25 areas of daily life in the NH to assess older people’s physical, psychological, social, and environmental needs. A distinction is made between met and unmet needs. Table 4 shows the outcomes of CANE studies and gives an overview of the five most frequently mentioned needs in each of these ten studies. Eight studies reported both unmet and met needs [29,34,44,51,54,59,64,68]. One study reported only unmet needs [53], and the study by van der Ploeg et al. [65] reported the sum of met and unmet needs differentiated between residents with dementia, residents without dementia, and relatives. Looking at the results without including the study by van der Ploeg et al. [65], the five most frequently mentioned met needs are in the areas of food, household skills, physical health, accommodation, and self-care. In comparison, the five most frequently unmet needs are in the areas of daytime activities, psychological distress, company, eyesight/hearing, and memory. Some of the five most frequently identified needs that residents have according to CANE studies were also highlighted by the analysis of the 41 other studies. These include the following needs in the area of unmet needs: daytime activities, psychological distress, and company. The met needs, which have also been addressed by the other studies, are as follows: food, physical health, and accommodation. Additional needs identified through the CANE studies that have not been mentioned in the previous analysis are household skills and self-care in the area of met needs and memory and eyesight/hearing related to unmet needs.

## 4. Discussion

The objective of this scoping review was to identify the wishes and needs of NH residents. The results show numerous needs that were mapped to 12 themes. In 35 studies, residents were interviewed; in 12 studies, residents and proxies were interviewed; and only proxies were interviewed in four studies. This shows that residents can be aware of perceived needs and wishes and can communicate them. This is valid not only for residents without cognitive impairment [69], but also for residents with dementia [11]. Studies show that third-party assessments of needs sometimes differ from what NH residents report [20,35,44,46,54,65]. This finding is especially important for residents with dementia, as needs elicitation for these individuals is often only collected through a proxy survey [11]. It is essential to directly survey NH residents, including residents with dementia, about their wishes and needs. Interviewing proxies can provide additional and helpful information, but is not a substitute for speaking directly with the affected resident.

The scoping review results further indicate that wishes and needs on specific topics differ between individual residents. For example, some would like to receive life-sustaining measures, while others reject them. This high degree of individuality and complexity must be considered in assessing needs. The wishes and needs should be recorded with the individual residents in private conversations, reflected on repeatedly, and the way they are dealt with should be adjusted if necessary. This requires time, expertise, and willingness. Often, there is a lack of human resources to ensure this task is completed. Complaints about a shortage of skilled workers and high workloads in NHs are frequent. [70,71]. These circumstances can lead to less quality in care and can make it difficult to have an individualized approach to residents [72]. Assessment tools, such as the PELI-NH or CANE questionnaire, can be helpful in conducting a comprehensive needs assessment. Such tools can provide clues to existing needs and wishes and present an overview. The CANE questionnaire, for example, does not address all the areas in which NH residents experience needs. Topics that are relevant for residents according to the present study, such as death/dying, autonomy, interaction of nursing staff with residents, and religion/spirituality, are not surveyed by this instrument. When caregivers or other persons refer to the CANE questionnaire in order to assess needs, they should be aware of this. Accordingly, in-depth and recurring interviews with residents are indispensable to consider the high complexity and individuality of wishes and needs. Only in this way can the results be validated and unmet needs can be discovered.

Themes of high relevance seem to be the following, as they were mentioned frequently and in multiple studies: “autonomy, independence, choice, and control”, “death, dying, and end-of-life”, and “medication, care, treatment, and hygiene”. Notably, needs cannot be categorized in a blanket way in which some needs are of higher importance than others. For example, needs in the nursing area may weigh the heaviest for some residents, while others consider the needs for autonomy and self-determination to be most important. 

Older adults are aware of their wishes and needs, but in many cases they do not communicate them [73]. Sometimes, when asked about their wishes and needs, residents report that they do not wish for anything because nothing would change anyway. The reason for this seems to be an experienced lack of respect for their wishes. For residents who have the feeling that their personal and subjective wishes and needs are not heard and that addressing them does not lead to any change, communicating their needs does not make sense [62,69]. As another reason for non-communication, older adults in home care state that they do not want to be a burden to anyone, and they do not want to complain about the age-related ailments that are common for them [73]. In these situations, caregivers should treat residents with appreciation and respect. It is important to schedule sufficient time to talk about wishes and needs. It is also important to take residents seriously and show them that expressing their wishes and needs will lead to positive changes in their lives by addressing them. The patronizing communication that often occurs on the part of NH staff may also contribute to NH residents not always openly communicating their wishes and needs, as satisfaction with such interactions can be low [74]. Further, the use of elderspeak due to stereotypical expectations of NH residents’ communication skills can lead to residents not feeling understood or respected and, as a result, they tend to be quiet and accept things without argument [75,76]. As a result, non-communicated needs go unrecognized and, accordingly, unmet. Communication training or person-centered interventions for caregivers could contribute to improved caregiver–patient communication, which could lead to more openness on the part of the residents and, consequently, fewer unmet residents’ needs [77,78].

Shared decision making was a frequently mentioned need. However, sometimes less is more. The study by Reed et al. [79] shows that older people prefer to have fewer options from which to choose than younger people. This suggests that some NH residents may be overwhelmed by too many options. NH staff should individually ask residents whether they prefer to choose from reduced options in some areas of their lives. 

The present study has some limitations. First, it must be said that the concepts of “wishes” and “needs” are very complex, and there is no common definition [80]. This can lead to the fact that all researchers involved understand something different by the concept under investigation. A definition was created and applied throughout to prevent this from happening and to ensure consistent study inclusion, data extraction, and analysis. Further, the 51 included studies are diverse in research design, study population, and objectives. For example, there are studies that surveyed residents as well as studies that surveyed proxies. Some studies focused on residents with dementia, while others focused on residents without cognitive impairment, or on unbefriended residents. The research focus was not primarily on wishes and needs in all studies. Constructs such as quality of life, dignity, or thriving were sometimes of substantial research interest. However, relevant wishes and needs were mentioned in the survey on these constructs, which were analyzed here. In the analysis of the quantitative studies, only the five most frequently mentioned wishes and needs were recorded in each case. The disadvantage here is that some wishes and needs were not recorded as a result. As qualitative studies do not include frequencies and therefore no ranking, all needs and wishes were extracted in these, which can lead to an overweighting of the qualitatively surveyed wishes and needs. Further, only studies in English and German were included. This can be explained by the language skills of the researchers but presents the possibility that relevant studies were not included. Consequently, the results only represent an overview of possible wishes and needs as stated by residents or their proxies. In no way do the results claim to be exhaustive of all wishes and needs of NH residents.

Among this study’s strengths is a very extensive literature search of 12 databases that was conducted. Additionally, the evidence examined is extensive, with 51 studies, as demonstrated by the high richness of results.

## 5. Conclusions

Twelve topics were identified to which the wishes and needs of NH residents can be assigned. This reflects the high complexity and diversity of the needs and wishes of the heterogeneous group of NH residents. 

For many NH residents, the NH represents the last phase of life before death. Residents should live a contented and fulfilling life in the home. Essential to achieving satisfaction is the fulfillment of individual wishes and needs. A comprehensive needs assessment on resident wishes and needs should take place in NHs. Speaking directly with the residents is essential to success.

The results of this study provide an evidence-based framework that can serve as a basis for holistic and person-centered care in NHs.

## Figures and Tables

**Table 1:** Search Terms in English and German.

	Population	Concept	Context
**English Search Terms**	elder OR elder people OR elder person OR senior OR old people OR old adult OR old age OR home resident OR resident OR age	need OR request OR wish OR preference OR concern OR demand OR unmet need	nursing home OR residential home OR retirement home OR long-term care home OR special-care home OR old people home OR home for the aged OR residential care OR long-term care
**German Search Terms**	Ältere Mensch ODER Ältere ODER Senior ODER Bewohner ODER Heimbewohner ODER Pflegebedürftige ODER Betagte ODER Hochbetagte ODER Hochaltrige	Bedürfnis ODER Wunsch ODER Wünsche	Altenheim ODER Altersheim ODER Pflegeheim ODER stationäre Pflege ODER stationäre Dauerpflege ODER stationäre Wohn ODER Alteneinrichtung ODER Pflegeeinrichtung ODER Senioreneinrichtung ODER stationäre Einrichtung

**Table 2:** Study Summary.

Author, Year	Country	Aim	Population	Type, Design Methods	Key Findings
Abbott et al. (2018) [18]	USA	To examine what the most and least important preferences of NH residents are and if those preferences change over time	N = 255 residents (68% women); M = 81 years (SD = 11)	Quantitative; A longitudinal questionnaire study using the PELI-NH questionnaire	Of 72 preferences, 16 were rated as very or somewhat important by 90% or more of residents; Key resident factors are taking care of their belongings, that staff show respect, that staff show they care about the residents, that they have regular contact with family, and that they can do what helps them feel better when they are upset
Bangerter et al. (2016) [19]	USA	To assess older adults’ preferences	N = 337 residents from 35 facilities (71% women); M = 81 years (SD = 11)	Qualitative and quantitative; A cross-sectional study using the PELI-NH questionnaire and open-ended questions	Residents identified preferences for interpersonal interactions, coping strategies, personal care, and healthcare discussions; Residents indicate that professional care is essential to them, that they are greeted by name by nursing staff, that their bathroom needs are met, and that they have a comfortable bed
Ben Natan (2008) [20]	Israel	To examine the congruence between needs identified as significant by older adults in comparison with caregivers and elders’ families	N = 182 (44 residents, 44 relatives, 94 caregivers). Residents: 60% women; M = 77 years (SD = 11). Relatives: M = 55 years. Professional Caregivers: M = 40 years	Quantitative; A cross-sectional questionnaire study	Key resident factors are skilled mental/emotional support, independence, a trustful relationship to the nurses, family visits, and a clean environment; Nurses alternatively attribute the most significant importance to resident values and personal attitudes, provision of competent physical care, competent spiritual support, social life, and institutional requirements; Families attribute the most significant importance to the provision of information and family involvement
Bergland and Kirkevold (2008) [21]	Norway	To describe NH residents’ perceptions of the significance of relationships with peer residents to their experience of thriving	N = 26 residents (77% women); range = 74–103 years	Qualitative; An exploratory cross-sectional study with open-ended interviews and field observation	NH residents have varied wishes regarding interaction with other residents, including the following needs: having personal and social contact with peer residents; being visited by relatives; keeping frequent and close contact with the family
Bollig et al. (2016) [22]	Norway	To study the views of cognitively able residents and relatives on advance care planning, end-of-life care, and decision making in NHs	N = 43 (25 residents, 18 relatives). Residents: 56% women; M = 87 years. Relatives: M = 68 years	Qualitative; An exploratory cross-sectional study with in-depth interviews with NH residents and focus group interviews with relatives	The main findings of this study were the different views on death and dying, decision making, and advance planning of residents and relatives; End-of-life wishes would relate to pain relief, companionship, dying as a relief, natural death, and life-prolonging treatments; Most residents indicate that their loved ones should decide if they are incapable of deciding themselves
Chabeli (2003) [23]	South Africa	To explore and describe the health needs of the aged living permanently at a NH in Gauteng	N = 27 residents (78% women); M = 74 years; range = 60–100 years	Qualitative; An exploratory cross-sectional study with focus groups	Three main data sets emerged: physical health needs; unmet psychological needs; the need for a healthy social relationship
Chamberlain et al. (2020) [24]	Canada	To identify unbefriended resident characteristics and their unmet care needs	N = 42 (39 Long term care staff, 3 public guardians)	Qualitative; An exploratory cross-sectional study with semi-structured interviews	Unbefriended residents have limited financial resources, often due to long-term disability or previous lifestyle leading to unmet needs such as difficulty obtaining personal care items due to limited financial resources and external social supports
Chan and Pang (2007) [25]	China/Hong Kong	To understand quality of life concerns and end-of-life care preferences of older people living in long-term care facilities in Hong Kong	N = 287 residents. Frail group: N = 164 (79% women); M = 84 years. Non-frail group: N = 123 (76% women); M = 82 years	Quantitative; A cross-sectional questionnaire study	Residents want stakeholder involvement with relatives and the attending physician to be involved in treatment decisions; The physician’s opinion is considered the most crucial, followed by the resident’s opinion, and the family’s opinion, respectively
Chu et al. (2011) [26]	China/Hong Kong	To describe the knowledge and preferences of Hong Kong Chinese older adults regarding advance directives and end-of-life care decisions	N = 1600 residents from 140 facilities (66% women); M = 82 years (SD = 7)	Quantitative; A cross-sectional questionnaire study	Majority preference for cognitively normal Chinese NH residents: have an advance directive; want to be informed of the diagnosis if they had terminal diseases; one-third of them would prefer to die in the NH
Chuang et al. (2015) [27]	Taiwan	To explore the older NH residents’ care needs from their own perspectives	N = 18 residents (17% women); M = 81 years	Qualitative; An exploratory cross-sectional study with in-depth interviews	Six themes relating to the care needs were generated, including body, environment, economics, mind, preparation for death, and social support care needs
Cooney et al. (2009) [28]	Ireland	To identify the determinants of quality of life for older people living in residential care, including exploration of mediating factors at personal and institutional levels, and to construct a model of these	N = 101 residents aged over 65 years (33% women)	Qualitative; A cross-sectional study with semi-structured interviews	Needs and wishes would have an impact on the quality of life of the residents; Quality of life factors can be found in the areas of control and involvement, privacy, connectedness and social relationships, and activities
Ferreira et al. (2016) [29]	Portugal	To describe the needs of an institutionalized sample and to analyze its relationship with demographic and clinical characteristics	N = 175 residents (90% women); M = 81 years (SD = 10); MMSE: M = 22	Quantitative; A cross-sectional questionnaire study using the CANE questionnaire	The met needs are in the fields: household skills, food, physical health, drugs, and money; The unmet needs are in the fields: daytime activities, eyesight/hearing, psychological distress, company, and memory
Franklin et al. (2006) [30]	Sweden	To explore the views on dignity at the end-of-life of older adults living in NHs in Sweden	N = 12 residents aged over 85 years (83% women)	Qualitative; A longitudinal study with semi-structured interviews	Multiple themes related to dignity in the NH were exposed: the unrecognizable body; fragility and dependence; and inner strength and a sense of coherence; Within these themes, wishes and needs could be identified as being seen or treated in a personal way; being visited by relatives; having conversations; finding meaning in everyday life; and being involved
Funk (2004) [31]	Canada	To describe decision-making preferences among residents of long-term care facilities	N = 100 residents (82% women); M = 85 years	Quantitative; A cross-sectional verbal questionnaire study	Residents with higher levels of formal education, a greater number of chronic conditions, and greater confidence in the value of their input tend to prefer more active involvement in decision making: 65% of residents want to decide independently when to go to bed; 46% want to decide advanced directives independently; 50% prefer some form of joint decision making
Gjerberg et al. (2015) [32]	Norway	To explore NH patients’ and next-of-kin’s experiences with and perspectives on end-of-life care conversations, information, and shared decision making	N = 68 (35 residents, 33 relatives). Residents: 77% women; M = 86 years; range = 68–98 years	Qualitative; An exploratory cross-sectional study with semi-structured interviews with NH residents and focus group interviews with relatives	Most relatives want conversations at the end-of-life, while the patients’ opinions vary; With some exceptions, patients and relatives want to be informed about the patient’s health condition; Many residents and relatives want to be involved in the decision-making process; Regarding the final treatment decision, the patients’ opinion varies: some patients want to leave the decisions entirely to the staff; few want to have the full responsibility
Goodman et al. (2013) [33]	UK	To explore how older people with dementia discuss their priorities and preferences for end-of-life care	N = 18 residents (72% women); M = 85 years	Qualitative; An exploratory cross-sectional study with guideline-based expert interviews	Three linked themes that had relevance for thinking and talking about the end-of-life were identified as “dementia and decision-making”, “everyday relationships”, and “place and purpose”; The preferences and priorities of the residents affect the everyday relationships and the significance of purpose and place; The residents specify diverse wishes and needs regarding those themes
Hancock et al. (2006) [34]	UK	To identify the unmet needs of people with dementia in care and the characteristics associated with high levels of needs	N = 238 professional caregivers as proxies. Residents: M = 87 years (SD = 7)	Quantitative; A cross-sectional questionnaire study using the CANE questionnaire	The met needs are in the fields: household skills, accommodation, self-care, money, and food; The unmet needs are in the fields: daytime activities, psychological distress, memory, eyesight/hearing, and behavior
Heid et al. (2017) [35]	USA	To examine the concordance in reports of importance ratings of everyday preferences for residents	N = 85 dyads of a resident and a family member; Residents: 72% women; M = 82 years (SD = 10)	Quantitative; A cross-sectional study using the PELI-NH questionnaire	Residents indicate the most important needs are spending time with family, respectful staff, choosing who is involved in discussions about care, choosing how to care for the mouth, choosing medical professionals, and caring caregivers
Heid et al. (2020) [36]	USA	To examine the impact of demographic and clinical characteristics of NH residents on the stability of their preferences over time	N = 255 residents (68% women); M = 81 years (SD = 11)	Quantitative; A longitudinal questionnaire study using the PELI-NH questionnaire	Residents indicate the following as essential needs: keeping the room at a certain temperature, caring for personal belongings, doing what helps one to feel better when you are upset, choosing how often to bathe, and choosing how to care for the mouth
Heusinger and Dummert (2016) [37]	Germany	To investigate residents’ gender-specific perception of life and care in NH	N = 20 residents (50% women); range = 72–93 years	Qualitative; A exploratory cross-sectional study with guideline-based interviews	In the area of personal hygiene, both universal and gender-specific needs were identified; The desire for respect for dignity and privacy was found across all genders; Universal across gender is the need for meaningful communication and mindful relationship building; Gender-specific wishes relate to the gender of the persons who assist with or perform personal care
Housen et al. (2009) [38]	USA	To evaluate a draft preference assessment tool (draft-PAT) designed to replace the current Customary Routine section of the Minimum Data Set (MDS) for NH	N = 198 residents (9% women); 72% no cognitive impairment	Quantitative; A verbal questionnaire study with two surveys within 72 h	This study finds that NH residents can reliably report their preferences; The preferences lie in the areas of activities, autonomy, functional competence, spiritual well-being, privacy, and security
Kane et al. (1997) [39]	USA	To examine the importance that NH residents and nursing assistants ascribed to control and choice over everyday issues, the satisfaction of residents with their control and choice over these issues, and the nursing assistants’ impressions of the extent to which control and choice exist for NH residents	N = 135 residents (69% women); M = 79 years	Qualitative and quantitative; cross-sectional in-person interviews using semi-structured interview protocols with both fixed-choice and open-ended questions	Cognitively intact NH residents attach importance to choice and control over matters such as bedtime, rising time, food, roommates, care routines, use of money, use of the telephone, trips out of the NH, and initiating contact with a physician; Nursing assistants view such control as important to residents
Klemmt et al. (2020) [40]	Germany	To explore wishes and needs, such as existing and preferred communication processes, of residents and relatives regarding medical and nursing planning at the end-of-life	N = 32 (24 residents, 8 relatives). Residents: 79% women; M = 89 years (SD = 7); range = 74–98 years. Relatives: 63% women; M = 56 years (SD = 3); range = 52–59 years	A qualitative; cross-sectional multicentric study with guideline-based interviews	Residents at the end-of-life primarily express wishes and needs regarding their health and social situation, for example: the desire to maintain or improve their current state of health and to be active and mobile; well-being of relatives and loved ones
Kurkowski et al. (2018) [41]	Germany	To identify the wishes of residents for their dying who live in a residential or NH	N = 9 residents (89% women); M = 88 years	Qualitative; An exploratory cross-sectional study with guideline-based expert interviews	Residents express, among other things, the following wishes: not to receive life-prolonging measures, not to have pain, not to need care or be bedridden, to receive affection while dying, and to find forgiveness and reconciliation, as well as to die peacefully in the NH; The study shows that residents are thinking about dying and/or death, have desires for their dying, and are also willing to talk about it
Levy-Storms (2002) [42]	USA	To compare three interview methodologies to assess NH residents’ unmet needs regarding activity of daily living care	N = 70 residents (82% women); M = 81 years; range = 79–104 years	Qualitative and quantitative; A cross-sectional study using a questionnaire and open-ended questions	The care of activities of daily living includes diverse wishes and needs on the part of the residents: teamwork between residents and staff, continuous and competent care; sensitivity on the part of the staff is important for the residents to implement successful care
Man-Ging et al. (2015) [43]	Germany	To report unaddressed psychosocial and spiritual needs among older people living in residential and NHs in Bavaria in southern Germany	N = 112 residents (76% women); M = 83 years (SD = 8)	Quantitative; A cross-sectional questionnaire study using the Spiritual Needs Questionnaire (SpNQ)	The ranking of specific needs shows a wide range of relevant needs: the need for prayer and relationships are of high importance; the most substantial needs are to “pray for yourself”, followed by to “reflect on one’s past life”, to “participate at a religious ceremony”, to “turn to a higher presence”, to “plunge into beauty of nature”
Mazurek et al. (2015) [44]	Poland	To analyze the complex needs of NH residents in different Polish cities from different perspectives and to explore the unmet need associations of health-related factors	N = 300 residents (79% women); M = 83 years (SD = 6); MMSE: M = 15	Quantitative; A cross-sectional questionnaire study using the CANE questionnaire	The met needs are in the fields: food, physical health, household skills, accommodation, and mobility/falls; The unmet needs are in the fields: company, psychological distress, eyesight/hearing, intimate relationships, and daytime activities
Michelson et al. (1991) [45]	USA	To elicit medical care preferences from NH residents	N = 44 residents (73% women); M = 84 years (SD = 6); range = 72–96 years	Quantitative; A cross-sectional study using case vignettes	Overall results show that study participants are opposed to aggressive medical treatment except where intervention would alleviate pain or result in greater patient comfort or safety; This reaction is particularly pronounced when participants are confronted with questions concerning the treatment of debilitated elderly patients with dementia
Milke et al. (2006) [46]	USA and Canada	To compare families, direct caregivers, and other staff and volunteers on their perception of the degree to which residents’ needs were being met	N = 277 (93 professional caregivers, 25 non-nursing staff, 25 volunteers, 134 family members and nearby persons)	Quantitative; A cross-sectional questionnaire study	Resident needs are in the areas of physical equipment, room personalization, physical care, meals, daily living behaviors, problem behaviors, medication, social activities, social and emotional support, physicians, caregivers, family, and volunteers
Milte et al. (2018) [47]	Australia	To elicit consumer preferences and their willingness to pay for food service in NH	N = 121 (43 residents, 78 family members). Residents: 66% women; M = 69 years (SD = 15)	Quantitative; A cross-sectional discrete choice experiment	Participants’ preferences are influenced by taste, choice in portion size, timing of meal, visual appeal, and additional cost; Above all, residents want delicious food at fixed times, to be involved in menu planning, and to be allowed to take their meals at their leisure
Mroczek et al. (2013) [48]	Poland	To analyze psychosexual needs of NH residents in Poland	N = 85 residents (60% women); M = 74 years (SD = 11)	Quantitative; A cross-sectional questionnaire study	The most essential psychosexual needs include conversation, tenderness, emotional closeness (empathy and understanding), sexual contact, and physical closeness
Nakrem et al. (2011) [49]	Norway	To describe the NH residents’ experience with direct nursing care, related to the interpersonal aspects of quality of care	N = 15 residents (60% women); range = 75–96 years	Qualitative; An exploratory cross-sectional study with in-depth interviews	Residents emphasize the importance of nurses acknowledging their individual needs, which includes the need for general and specialized care, health promotion and the prevention of complications, and prioritizing the individuals; Psychosocial well-being is a major issue, and the residents express an important role of the nursing staff helping them to balance the need for social contact and to be alone, and preserving a social network
Ni et al. (2014) [50]	China	To describe Chinese NH residents’ knowledge of advance directive and end-of-life care preferences	N = 467 residents (60% women); M = 77 years (SD = 9)	Quantitative; A cross-sectional questionnaire study	More than half of the residents would receive life-sustaining treatment if they sustained a life-threatening condition; Most residents nominate their eldest son or daughter as their proxy; More than half wanted to live and die in their present NHs
Nikmat and Almashoor (2015) [51]	Malaysia	To identify the needs of people with cognitive impairment living in NHs and factors associated with higher level of needs	N = 110 residents (50% women); M = 72 years (SD = 8); MMSE: M = 5	Quantitative; A cross-sectional questionnaire study using the CANE questionnaire	The met needs are in the fields: accommodation, looking after home, food, money, and self-care; The unmet needs are in the fields: intimate relationships, company, daytime activities, caring for another, and memory
O’Neill et al. (2020) [52]	UK	To explore the residents’ experiences of living in a NH, during the 4- to 6-week period following the move	N = 17 residents (59% women); M = 83 years	Qualitative; An exploratory cross-sectional study with guideline-based interviews	Three main themes in the initial implementation phase in the NH could be identified in relation to wishes and needs: wanting to connect, wanting to adapt, and wanting to re-establish links with family and home
Orrell et al. (2007) [53]	UK	To reduce unmet needs in older people with dementia in residential care compared to a ‘care as usual’ control group	N = 238 residents; intervention group: 76% women; M = 87 years (SD = 7). Control group: 83% women; M = 86 years (SD = 8)	Quantitative; A cross-sectional questionnaire study (single-blind, multicenter, cluster randomized controlled trial cRCT) using the CANE questionnaire	The unmet needs are in the fields: daytime activities, memory, eyesight/hearing, company, and psychological distress
Orrell et al. (2008) [54]	UK	To compare the ratings of needs of older people with dementia living in NH, as assessed by the older person themselves, a family caregiver, and the staff	N = 468 (238 professional caregivers as proxies, 149 residents, 81 family caregivers)	Quantitative; A cross-sectional questionnaire study using the CANE questionnaire	The met needs are in the fields: food, accommodation, household skills, mobility/falls, and self-care; The unmet needs are in the fields: daytime activities, company, psychological distress, eyesight/hearing, and information
Paque et al. (2018) [55]	Belgium	To explore general feelings among NH residents, with a specific interest in loneliness to develop strategies for support and relief	N = 11 residents (64% women); M = 84 years; range = 74–92 years	Qualitative; An exploratory cross-sectional study with face-to-face interviews	Loneliness is more than being alone, among others; The residents’ unfulfilled need for meaningful relationships plays a crucial role in feelings of loneliness
Reynolds et al. (2002) [56]	USA	To describe the palliative care needs of dying NH residents during the last three months of life	N = 176 professional caregivers and relatives of 80 deceased residents. Residents at time of death: 61% women; M = 82 years; range = 54–99	Quantitative; An exploratory retrospective cross-sectional study and verbal questionnaire survey	A total of 90% of the residents died in the NH rather than in a hospital; Most deaths were preceded by orders to withhold resuscitation and other treatments; Respondents believed residents needed more treatment than they received for emotional symptoms, personal cleanliness, and pain
Riedl et al. (2013) [57]	Austria	To explore what NH residents need in their first year after having moved into a NH to maintain their identity and self-determination	N = 20 residents (75% women); M = 82 years; range = 71–93 years	Qualitative; An exploratory cross-sectional study with problem-centered interviews	The participants of this study resist against having decisions taken away from them and fight for their independence and identity; To be able to cope with these strains, they need the help of family members, professionals, and identity-forming conversations in new social networks in the NH
Roberts et al. (2018) [58]	USA	To describe the overall resident preferences, the variation in preferences across items, and the variation in preferences across residents	N = 244.718 residents from 14.492 facilities (65% women); M = 81 years (SD = 8)	Quantitative; A cross-sectional questionnaire study	Most residents rate all 16 preferences of the Minimum Data Set 3.0 (MDS) Preference Assessment Tool (PAT) important (notable variation across items and residents); Involvement of family in care and individualizing daily care and activities are rated important by the largest proportion of residents
Roszmann et al. (2014) [59]	Poland	To describe the met and unmet needs of NH residents and to learn about the living conditions of older people living in institutions, focusing on their various needs	N = 98 residents (74% women); M = 81 years; range = 63–93 years;	Quantitative; A cross-sectional questionnaire study using the CANE questionnaire	The met needs are in the fields: drugs, physical health, self-care, household skills, and continence; The unmet needs are in the fields: accommodation, memory, food, psychological distress, and company
Schenk et al. (2013) [60]	Germany	To identify dimensions of life that NH residents perceive as having a particular impact on their overall quality of life	N = 42 residents (79% women)	Qualitative; A cross-sectional study with semi-structured interviews	Wishes and needs that the study evaluated in relation to quality of life relate to the areas: social contacts, self-determination and autonomy, privacy, activities, feeling at home, security, and health
Schmidt et al. (2018) [61]	Germany	To identify the needs of people with advanced dementia in their final phase of life and to explore the aspects relevant to first recognize and then meet these needs	N = 30 residents (77% women); M = 84 years; range = 75–93 years	Qualitative; An exploratory cross-sectional study with focus groups, interviews, and field observation	Data analyses generate 25 physical, psychosocial, and spiritual needs divided into ten categories. Physical needs are classified as follows: “food intake”, “physical well-being”, and “physical activity and recovery”; Categories of psychosocial needs are classified as follows: “adaptation of stimuli”, “communication”, “personal attention”, “participation”, “familiarity and safety”, as well as “self-determination”. Spiritual needs address “religion”
Sonntag et al. (2003) [62]	Germany	To examine the wishes of NH residents concerning their life situation in the NH	N = 1656 residents	Qualitative; An exploratory cross-sectional study with one open question	The analyses of residents’ wishes lead to major domains such as the quality of care, interpersonal contact, architecture and organization of the house, diversification, financial support, as well as themes such as health and death and the wish to leave the NH
Strohbuecker et al. (2011) [63]	Germany	To explore the palliative care needs of NH residents in Germany who had not yet entered the dying phase	N = 9 residents (78% women); M = 87 years	Qualitative; An exploratory cross-sectional study with face-to-face interviews	The residents describe multidimensional needs, which are categorized as “being recognized as a person”, “having a choice and being in control”, “being connected to family and the world outside”, “being spiritually connected”, and “physical comfort”. They emphasize their desire to control everyday matters
Tobis et al. (2018) [64]	Poland	To investigate the patterns of needs in older individuals living in long-term care institutions using the CANE questionnaire	N = 306 residents (75% women); M = 83 years (SD = 6); MMSE: M = 23	Quantitative; A cross-sectional questionnaire study using the CANE questionnaire	The met needs are in the fields: looking after home, food, physical health, accommodation, and self-care; The unmet needs are in the fields: company, psychological distress, eyesight/hearing, intimate relationships, and daytime activities
van der Ploeg et al. (2013) [65]	Netherlands	To compare the number and type of needs of people with and without dementia in residential care in the Netherlands	N = 187 residents (75% women); M = 87 years; range = 72–98 years	Quantitative; A cross-sectional questionnaire study using the CANE questionnaire	The sum of met and unmet needs of residents with dementia are in the fields: household skills, food, mobility/falls, self-care, and physical health; The sum of met and unmet needs of residents without dementia are household skills, mobility/falls, food, accommodation, and physical health; The sum of met and unmet needs according to the relatives are food, household skills, accommodation, mobility/falls, and self-care
van der Steen et al. (2011) [66]	Netherlands	To assess preferences relevant to dementia patients, pilot-testing the ‘Preferences About Death and Dying’ instrument for palliative care	N = 30 residents (93% women); 60% severe dementia; M = 89 years (SD = 6)	Quantitative; A cross-sectional questionnaire study	Pain under control, comfortable breathing, and dignity are most important (note no one is rating these as unimportant); A condition during the dying process and the place of death; Residents do not want to receive any life-sustaining treatments and hope to have recognized meaning and purpose at the end-of-life
van Oorschot et al. (2019) [67]	Germany	To explore NH residents’ desired place of death, living will, and desired care at end-of-life	N = 197 residents (72% women); M = 87 years; range = 59–98 years	Quantitative; An exploratory cross-sectional study and verbal questionnaire survey	Many residents wish to die in the NH because they view the NH as a place to die much more positively than is often discussed; Fewer residents want to die in hospital, followed by hospice and private household
Wieczorowska-Tobis et al. (2016) [68]	Poland	To evaluate the CANE questionnaire in assessing the needs of elderly individuals living in long-term care institutions in Poland	N = 173 residents (80% women); M = 83 years (SD = 6)	Quantitative; A cross-sectional questionnaire study using the CANE questionnaire	The met needs are in the fields: physical health, caring for another, mobility/falls, food, and continence; The unmet needs are in the fields: daytime activities, company, psychological distress, eyesight/hearing, and intimate relationships

Note: all values have been rounded to the nearest whole number for consistency; M stands for mean; SD stands for standard deviation.

**Table 3:** Explicit description of the themes.

Themes	Outcomes
(1) Activities, leisure, and daily routine	Recreational, enjoyable, meaningful, and person-specific activities; More and more diverse activities and events; Activities on special occasions and holidays; Being self-reliant and physically active in daily activities.
(2) Autonomy, independence, choice, and control	Maintaining independence, individuality, and self-determination, feeling autonomous; Having choice over life in the NH; Choosing who is involved in decision making; Consulting a physician; Having an advance directive, having a living will; Leaving the NH unaided; Moving out of the NH, deciding on discharge.
(3) Death, dying, and end-of-life	Dying in place of choice; Dying in state of choice; Dying alone or with company; Natural and quick death; Peaceful and dignified dying; Talking about death and dying, discussing wishes and preferences regarding dying; Making arrangements and being prepared for death; Wanting life to end, death as a relief; Physical closeness, human warmth, and assistance; Forgiveness and reconciliation; Respectful, open, and honest communication; Being touched or hugged by loved ones, having close relationships; Relief of pain and thirst, breathing comfortably; Receiving life-sustaining treatments or devices or not receiving it; More time-consuming and personal care; Being mobile and active at the end-of-life, making plans.
(4) Economics	Having financial resources, financial security, pocket money, more money; Paying the monthly NH fee.
(5) Environment, structural conditions, meals, and food	Enough space, larger rooms; Keeping the room at a certain temperature; Bed-comfort; Clean environment; Place to lock the things, taking care of the belongings; Personalizing the room; Better sanitary facilities; NH suitable for the elderly with physical impairments and disabilities; Flexible organization of processes; Staying in a familiar area; Separation of demented and non-demented residents; Nicely prepared, tasty, interesting, traditional meals and snacks; Food that meets the special diet needs; Set meal times; Being asked for the opinion regarding the menu plan; Assistance at meal times if required; Enough time to enjoy the meals.
(6) Health condition	Being informed about the health condition or not being informed; Maintaining the physical health and the cognitive abilities; Preventing a decline in functioning; Not becoming a nursing case; Maintaining the mobility; Restful sleep; Physical comfort.
(7) Medication, care, treatment, and hygiene	Assistance for activities of daily living; Adequate (medical) treatment, good pain management; More active care, fall prevention, and therapeutic services; Proper and clean equipment for treatments; Regular pad change; Good skin and hair care, good oral and personal hygiene; Professional monitoring of unpleasant effects of medication; Refusing medications and medical treatment; Professional, respectful, friendly, encouraging, sensitive, caring, and experienced staff; Close, trustful, and continuous relationship with staff; More nursing staff; Not being embarrassed or treated as a child by staff, not being perceived as troublesome or burden; Respect for personality, being taken seriously, being recognized as a person; Being greeted with the name by staff; Personal care provided by a nurse who is a woman when the resident is a woman herself.
(8) Peer relationship, company, and social contact	Personal, meaningful, and harmonious contact with peer residents and family; Being visited by relatives, having regular contact, having more time with relatives; Having someone to talk to during the day; Inter-generational contacts; Links between the community, the world outside, and the residential facility; Preserving the former social network.
(9) Privacy	Social and psychological privacy; Resting undisturbed; Spending time alone.
(10) Psychological and emotional aspects, security, and safety	Getting support for diverse psychological and emotional problems; Doing what helps you feel better when you are upset; Feeling warm, needed, and valued; Positive self-esteem, positive attitude; Remaining oneself, preserving the identity; Expressing emotions and one’s own will; Engaging with others, touching others/being touched; Being involved; Being understood; Taking each day as it comes and not worrying too much about tomorrow; Personal resilience and ways of coping with the situation; Finding meaning in everyday life, enjoying small things; Feeling of security; Access to prompt emergency care; Safety precautions in the NH; Being protected from self-injury and from being bothered by other residents.
(11) Religion and spirituality	Participating in religious rituals, services, and ceremonies; Following cultural or family customs; Being spiritually connected; Praying, turning to a higher presence; Reflecting on previous life; Plunging into beauty of nature.
(12) Sexuality	Conversations; Respect; Tenderness; Support in any situation; Giving and receiving emotional support.

**Table 4:** Outcomes CANE studies.

Study	Met Needs Top 5	Unmet Needs Top 5	
Ferreira et al. (2016) Portugal [29]	1. Household Skills 2. Food 3. Physical health 4. Drugs 5. Money	1. Daytime activities 2. Eyesight/hearing 3. Psychological distress 4. Company 5. Memory	
Hancock et al. (2006) UK [34]	1. Household skills 2. Accommodation 3. Self-care 4. Money 5. Food	1. Daytime activities 2. Psychological distress 3. Memory 4. Eyesight/hearing 5. Behavior	
Mazurek et al. (2015) Poland [44]	1. Food 2. Physical health 3. Household skills 4. Accommodation 5. Mobility/falls	1. Company 2. Psychological distress 3. Eyesight/hearing 4. Intimate relationships 5. Daytime activities	
Nikmat and Almashoor (2015) Malaysia [51]	1. Accommodation 2. Looking after home 3. Food 4. Money 5. Self-care	1. Intimate relationships 2. Company 3. Daytime activities 4. Caring for another 5. Memory	
Orrell et al. (2007) UK [53]	n.a.	1. Daytime activities 2. Memory 3. Eyesight/hearing 4. Company 5. Psychological distress	
Orrell et al. (2008) UK [54]	1. Food 2. Accommodation 3. Household skills 4. Mobility/falls 5. Self-care	1. Daytime activities 2. Company 3. Psychological distress 4. Eyesight/hearing 5. Information	
Roszmann et al. (2014) Poland [59]	1. Drugs 2. Physical health 3. Self-care 4. Household skills 5. Continence	1. Accommodation 2. Memory 3. Food 4. Psychological distress 5. Company	
Tobis et al. (2018) Poland [64]	1. Looking after home 2. Food 3. Physical health 4. Accommodation 5. Self-care	1. Company 2. Psychological distress 3. Eyesight/hearing 4. Intimate relationships 5. Daytime activities	
van der Ploeg et al. (2013) Netherlands [65] (Here presented the sum of met and unmet needs distinguished between residents with and without dementia and relatives as proxies)	Residents with dementia 1. Household skills 2. Food 3. Mobility/falls 4. Self-care 5. Physical health	Residents without dementia 1. Household skills 2. Mobility/falls 3. Food 4. Accommodation 5. Physical health	Relatives 1. Food 2. Household skills 3. Accommodation 4. Mobility/falls 5. Self-care
Wieczorowska-Tobis et al. (2016) Poland [68]	1. Physical health 2. Caring for another 3. Mobility/falls 4. Food 5. Continence	1. Daytime activities 2. Company 3. Psychological distress 4. Eyesight/hearing 5. Intimate relationships	
